# Autosomal dominant and autosomal recessive polycystic kidney disease: hypertension and secondary cardiovascular effect in children

**DOI:** 10.3389/fmolb.2023.1112727

**Published:** 2023-03-10

**Authors:** L. Lucchetti, M. Chinali, F. Emma, L. Massella

**Affiliations:** ^1^ Division of Nephrology, Department of Paediatric Subspecialties, Bambino Gesù Children’s Hospital, IRCCS, Rome, Italy; ^2^ Department of Cardiac Surgery, Cardiology and Heart Lung Transplant, Bambino Gesù Children’s Hospital (IRCCS), Rome, Italy

**Keywords:** ARPKD, ADPKD, hypertension, cardiovascular disease, children

## Abstract

Autosomal dominant (ADPKD) and autosomal recessive (ARPKD) polycystic kidney disease are the most widely known cystic kidney diseases. They are significantly different from each other in terms of genetics and clinical manifestations. Hypertension is one of the main symptoms in both diseases, but the age of onset and secondary cardiovascular complications are significantly different. Most ARPKD children are hypertensive in the first year of life and need high doses of hypertensive drugs. ADPKD patients with a very early onset of the disease (VEO_ADPKD_) develop hypertension similarly to patients with ARPKD. Conversely, a significantly lower percentage of patients with classic forms of ADPKD develops hypertension during childhood, although probably more than originally thought. Data published in the past decades show that about 20%–30% of ADPKD children are hypertensive. Development of hypertension before 35 years of age is a known risk factor for more severe disease in adulthood. The consequences of hypertension on cardiac geometry and function are not well documented in ARPKD due to the rarity of the disease, the difficulties in collecting homogeneous data, and differences in the type of parameters evaluated in different studies. Overall, left ventricular hypertrophy (LVH) has been reported in 20%–30% of patients and does not always correlate with hypertension. Conversely, cardiac geometry and cardiac function are preserved in the vast majority of hypertensive ADPKD children, even in patients with faster decline of kidney function. This is probably related to delayed onset of hypertension in ADPKD, compared to ARPKD. Systematic screening of hypertension and monitoring secondary cardiovascular damage during childhood allows initiating and adapting antihypertensive treatment early in the course of the disease, and may limit disease burden later in adulthood.

## 1 Background

Autosomal dominant polycystic kidney disease (ADPKD) and autosomal recessive polycystic kidney disease (ARPKD) are the most well-known cystic kidney diseases. They belong to the group of ciliopathies, but are significantly different from each other in terms of genetics and clinical manifestations. In a not too distant past, they were named “adult” and “infantile” polycystic kidney diseases, respectively. Nowadays, these terms have been abandoned because they do not describe accurately the natural history of the diseases. Most ARPKD children are hypertensive in the first year of life and need very early high doses of antihypertensive drugs. A significantly lower percentage of ADPKD patients develop hypertension during childhood, but this percentage is probably underestimated. The PROPKD score, which has been validated based on outcome measures, indicates that early onset hypertension (before the age of 35 years) is a risk factor for fast progression of chronic kidney disease (CKD). The purpose of this review is not to compare ARPKD with ADPKD, but to review of the available pediatric studies (Table 1 and Table 2) and experience-based observations on cardiovascular aspects of both conditions.

**TABLE 1:** Published literature on hypertension in ADPKD children.

	Patient	Age (mean ± SDS)	OBPM HTN (%)	ABPM HTN (%)	Non-dipper (%)
Ivy et al. (1995)	83	9.6 ± 0,5	13	-	-
Seeman et al. (1997)	32	12.3 ± 4.7	16	34	12.5
Seeman et al. (2003)	62	12.3 ± 4.3	-	35	-
Shamshirsaz et al. (2005)	199	13.3	36	-	-
Stergiou et al. (2005)	17	13.3 ± 2.9	47	-	-
Cadnapaporchai et al. (2008)	85	13.6 ± 0.8	33	-	-
Cadnapaporchai et al. (2009)	85	14 ± 1	33	-	-
Mekahili et al. (2010)	47	12.9 ± 5.1	15	-	-
Selistre et al. (2012)	52	10 ± 4	6	-	-
Massella et al. (2018)	295	11.5 ± 4.1	-	35	52
Marlais et al. (2019)	47	11	21		35
Seeman et al. (2021)	69	14.8 ± 4.7	38	-	-

Abbreviations: OBPM, Office Blood Pressure Measurement; ABPM, ambulatory blood pressure measurement; HNT, hypertension. Hypertension: defined as blood pressure >95th centile for age, height and gender or if in anti- HTN treatment.

**TABLE 2:** Published literature on hypertension in ARPKD children.

	Cole et al. (1987)	Kaplan et al. (1989)	Zerres et al. (1996)	Roy et al. (1997)	Rhona Capisonda et al. (2003)	Guay-Woodford and Desmond (2003)	Bergmann et al. (2005)	Dell et al. (2016)	Chinali et al. (2019)	Seeman et al. (2022)
Patients	17	55	115	52	31	166	186	22	27	36
Follow-up (y)	14	37	6	43	10	12	6	-	-	12
Age at diagnosis	1°y	42% < 1 m	51% < 1°m	85% ≤ 1°y	55% < 1°m	73% < 1°m	54% < 1°m	0,1°y (mean)	-	0,2°y (median)
		42% 1-12 m	23% 1-12°m	15% > 1°y	19% 1-12°m	11% 1-12°m	16% 1-12°m			
		16% > 1 y	26% > 1 y		26% > 1°y	16% > 1°y	30% > 1°y			
ESKD	29%	-	10%	33% by 15°y	16%	13%	29% by 10°y	-	21%	0%
HTN	100%	65%	71%	-	55%	65%	-	86%	74%	86% (ABPM)
Anti- HTN treatment	-	-	70%	39% by 1°y	55%	65%	76%	86%	63%	86%
				60% by 15°y						
Age start drug	-	-	180°d (mean)	-	50 % by 5°y	16°d (mean)	3°y (median)	-	-	-
					58% by 15°y					
Hyponatremia	-	54%	25%	-	10%	26%	-	-	-	-
Patient survival rate	1°y 88%	1 y 79%	1°y 89%	-	1°y 87%	1°y 79%	1°y 85%	-	-	-
		10 y 51%	3°y 88%		9°y 80%	5°y 75%	5°y 84%			
		15 y 46%					10°y 82%			
Death rate in 1°y	12%	24%	9%	26%	13%	8%	15%	-	-	-

Abbreviations: y, year; d, day; m, months; ESKD, end stage kidney disease; HNT, hypertension. Hypertension: defined as blood pressure >95th centile for age, height and gender or if in anti- HTN treatment.

## 2 Autosomal dominant polycystic disease (ADPKD)

### 2.1 Introduction

ADPKD is the most common genetic kidney disease in adulthood, characterized by multiple and bilateral kidney cysts. Progressive enlargement of cysts starts early in life, causing kidney volume enlargement, leading to progressive decline of kidney function and ultimately end stage kidney disease (ESKD), usually around 50–60 years of age, depending on the underlying genetic defect. The disease is caused by pathogenic variants in the *PKD1* gene, located on chromosome 16p13.3 and in the *PKD2* gene, located on chromosome 4q21, encoding for polycystin-1 (PC1) and polycystin-2 (PC2), respectively. *PKD1* is responsible for the more severe phenotype and is involved in approximately 85% of cases. Most cases managed by pediatrician are secondary to *PKD1* variants or to rare digenic conditions, since these manifest earlier in life compared to *PKD2* variants. Cyst formation requires mutations of both alleles in either *PKD1* or *PKD2* genes. Since every cell carries one germ line mutation (first hit), a second somatic mutation in the normal allele (second hit) is needed to lead to the formation of cysts from previously normal epithelial cells of renal tubules and biliary ducts. The phenotypic expression of the disease is variable. Several factors explain this variability. In particular, the phenotypic expression depends on the type of variant, ranging from hypomorphic to variants that cause complete loss of function, which impacts on the amount of residual functioning polycistin proteins. The lower the levels, the faster the cyst formation and growth (Fedeles et al., 2014).

ADPKD is a systemic disorder with many clinical manifestations, including hypertension, left ventricular hypertrophy (LVH), heart valve disease, hepatic cysts, urinary tract infections, proteinuria, hematuria, kidney stones, intracranial and extracranial aneurisms (Perrone et al., 2001; Ecder, 2013; Chapman et al., 2015; Gimpel et al., 2019). Cardiovascular manifestations are the main extra-renal complications of ADPKD. With improvements in renal replacement therapy and kidney transplantation over the past decades, cardiovascular complications have become the most common cause of morbidity and mortality in adult patients with ADPKD (Fick et al., 1995; Helal et al., 2012).

In the past two decades, considerable progresses have taken place in our knowledge of the pathogenesis and clinical manifestation of ADPKD in children. As pediatricians, we now know that ADPKD is not only an adult disease and that some renal and extra-renal manifestations may appear during childhood, albeit sometimes they remain underdiagnosed (Fick et al., 1994; Fick-Brosnahan et al., 2001; Cadnapaphornchai, 2013).

Hyperfiltration and hypertrophy of unaffected nephrons may mask renal impairment, preserving normal glomerular filtration for many years (De Rechter et al., 2017). Approximately 60% of ADPKD patients have kidney cysts by the age of 5 years (Gabow et al., 1997). A subset of patients, termed Early Onset ADPKD (EO_ADPKD_), suffer from a severe form of the disease that manifest with significant symptoms between 1.5 and 15 years of age. These patients have early hypertension and rapid progression of CKD (MacDermot et al., 1998; Shamshirsaz et al., 2005). Another very small group of patients, named Very Early Onset ADPKD (VEO_ADPKD_), develop oligohydramnios and hyperechoic enlarged kidney *in utero* (MacDermot et al., 1998). In these patients, two hypomorphic biallelic variants in the *PKD1* gene are often identified resulting in a very severe phenotype and their clinical picture mimic that of ARPKD (Bergmann, 2019).

### 2.2 Pathogenesis and prevalence of hypertension

Arterial hypertension is the most frequent initial manifestation in ADPKD and is observed in 50%–75% of adults (Chapman et al., 2010a). Since most patients become symptomatic in adulthood the vast majority of the literature on this subject has been produced in adult patients. Altogether, studies have shown that hypertension usually develops around 30–35 years of age (i.e., earlier than essential hypertension) and precedes almost always the onset of CKD. These studies have also shown that hypertension may accelerate progression towards ESKD (Ecder and Schrier, 2001; Chapman et al., 2010a) and promotes the development of LVH (Gabow et al., 1992; Fick et al., 1995).

The pathogenesis of hypertension in ADPKD is multifactorial, involving the renin-angiotensin-aldosterone system (RAAS), the sympathetic nervous system, and endothelial dysfunction (De Rechter et al., 2017; Krishnappa et al., 2017).

The activation of the RAAS system plays a fundamental role. Progressive increase in the size of cysts causes compression of renal arterioles, leading to localized ischemia and hypoxia, which, in turn, activates the RAAS system (Chapman et al., 1990; Loghman-Adham et al., 2004). RAAS activation may then stimulate the sympathetic nervous system, increasing plasma catecholamines to higher levels in comparison to those observed in patients with essential hypertension (Schrier, 2009).

In addition, studies on cells derived from renal cysts, renal mesangium, and from the muscular layer of renal arteries have shown increased synthesis of endothelin 1 and over-expression of the Endothelin Receptor Subtype A in ADPKD, contributing to the development of arterial hypertension and gradual loss of kidney function (Hocher et al., 1998; Krishnappa et al., 2017). Sodium retention and reduced nitric oxide synthesis also appears to play a role. Low or absent levels of *PKD1* and *PKD2* gene expression have been associated with reduced nitric oxide synthesis, which results in impaired vascular response to stress and activation of the RAAS system (De Rechter et al., 2017; Krishnappa et al., 2017).

Arguably, many adult nephrologists suggest patients to test their children for possible ADPKD only after the age of 18 years, since the disease usually becomes symptomatic during adulthood. Current guidelines on the subject are limited and this matter remains controversial. The experience gained in other renal conditions indicates that delayed treatment of hypertension, including during childhood, can increase significantly cardiovascular morbidity (Gimpel et al., 2019).

**FIGURE 1:**
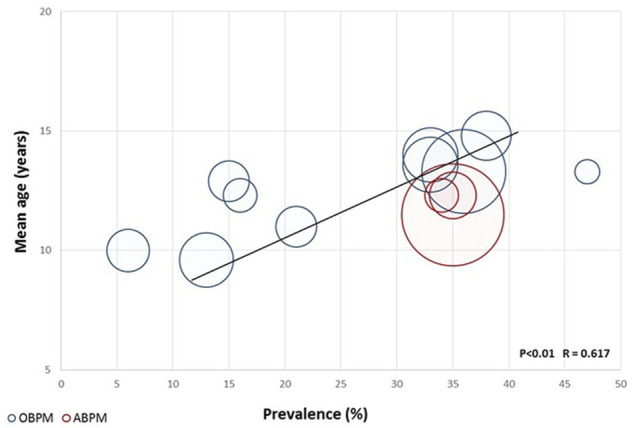
Prevalence of hypertension in ADPKD children according to age and measuring method.

As expected, the prevalence of hypertension in ADPKD increases with age in all studies, reaching >90% after the age of 50 years. Pediatric nephrology units that treat large number of children with ADPKD have observed a significant prevalence of hypertension in their patients (Figure 1).

Some early prevalence data were reported in 2010 by Mekhali et al., who observed a prevalence of hypertension of 15% in 47 patients (Mekahli et al., 2010). In the following years, other studies have reported prevalence data ranging 6%–44%, which are significantly higher than data reported in the general pediatric population (3%–5%) (Fick et al., 1994; Seeman et al., 2003; Kelleher et al., 2004; Cadnapaphornchai et al., 2008; Mekahli et al., 2010; Selistre et al., 2012; Cadnapaphornchai, 2013).

In 2016, Marlais performed a systematic review and a meta-analysis to better define the prevalence of hypertension in children and young adults with ADPKD. The analysis of 14 studies included 928 patients and revealed a mean prevalence of hypertension of 20%, even after removing the studies with a high risk of selection biases. A positive significant correlation between the mean age of the cohort and the prevalence of hypertension was observed across eleven of the fourteen selected studies. This meta-regression analysis confirms studies in adults, showing that onset of hypertension starts in a majority of patients during the third decade of life and increases rapidly thereafter. The authors also analyzed the prevalence of proteinuria in eight of the analyzed studies and report a prevalence of proteinuria in 20% of patients, although usually not severe (Marlais et al., 2016). No correlation was observed between the prevalence of proteinuria and the prevalence of hypertension. This meta-analysis had limitations due to the possible selection of more severe cases in tertiary centers that published their findings and to the fact that hypertension was not the primary outcome in any of the selected studies. In addition, cohorts were heterogeneous in terms of size, age, method for diagnosing hypertension (Marlais et al., 2016). One of the first study that used ambulatory blood pressure monitoring (ABPM) was published in 1997 and suggested that the prevalence of hypertension may have been be underestimated (Seeman et al., 1997). In most cases, hypertension was diagnosed based on office blood pressure measurements (OBPM) or home blood pressure measurements (HBPM), which may miss borderline forms of hypertension and do not allow diagnosing isolated nocturnal hypertension. ABPM represents the gold standard for detecting hypertension in children and in adults (Lurbe et al., 2016; Flynn et al., 2017). It is more accurate than office blood pressure in diagnosing hypertensive or pre-hypertensive patients (Stergiou et al., 2005; de Almeida et al., 2007) and allow detecting night-time hypertension or lack of nocturnal dipping (de Almeida et al., 2007; Brady et al., 2008). These latter two conditions may represent risk factors for the development of secondary organ damage and have been described in ADPKD patients. In addition, ABPM data correlate better with LVH than OBPM and HBPM (Valero et al., 1999; Brady et al., 2008). Only few studies have assessed hypertension in ADPKD children using ABPM (Zeier et al., 1993; Seeman et al., 1997; Seeman et al., 2003; Massella et al., 2018; Marlais et al., 2019).

In 2003, Seeman reported a prevalence of hypertension of 35% in 62 children evaluated by ABPM. In almost 30% of them, hypertension was nocturnal. Two-thirds of children with normal blood pressure by OBPM had hypertension by ABPM. A positive correlation between ABPM values, kidney volume, and the number of cysts was observed (Seeman et al., 2003).

In 2018, Massella published data from a European multicenter retrospective study on 310 ADPKD patients under the age of 18 with normal kidney function. The study showed that 21% of patients were hypertensive throughout the entire 24 h cycle. This rate increased to 35% when considering patients who were not found hypertensive by ABPM but were receiving treatment for hypertension. Nearly 18% of patients had isolated nocturnal hypertension and nearly half of patients lacked significant dipping at night. Logistic regression analysis showed a significant positive correlation between the number of cysts and daytime, night-time, and 24 h blood pressure values. Kidney length was significantly associated with night-time and isolated nocturnal hypertension (Massella et al., 2018).

Recently, Seeman et al. published a longitudinal study conducted on 69 ADPKD patients and 40 healthy subjects with an average follow-up of 6.3 years. Their data show an increase in the prevalence of hypertensive patients from 20% at the beginning of the study to 38% at the end of the study. During the observation period, there was no significant decline in kidney function or increase in proteinuria, but a significant increase in kidney size and in the number of renal cysts (Seeman et al., 2021) (Summary of the literature in Table 1).

### 2.3 Cardiovascular consequences of hypertension

The heart is the main target organ of hypertension in terms of secondary damage.

From the adult literature, we have learned that arterial hypertension can causes LVH and arteriosclerosis with an increase in cardiovascular mortality. Hypertension is not the only factor that induces LVH. Other factors, such as anemia, increased body mass index, excessive sodium intake, and increased activity of the RAAS system may also be involved (Gabow et al., 1992; Fick et al., 1995). Studies on adult patients with ADPKD have evaluated LVH by ultrasound or magnetic resonance imaging. Despite limitations due to the use of different instrumental techniques and different formulas for calculating the left ventricular mass index (LVMi), all these studies are in agreement in showing that the LVMi correlates with blood pressure values, kidney function, and total kidney volume (TKV) (Chapman et al., 1997; Ecder, 2013; Kuo and Chapman, 2020).

Several adult studies have also shown that intensive blood pressure control can reduce LVH and cardiovascular morbidity (Schrier et al., 2002; Chapman et al., 2010b; Schrier et al., 2014; Torres et al., 2014). In particular, the HALT-A study has shown that intensive blood pressure control (target 95–110/60–75 mmHg) in patients with ADPKD and relatively preserved kidney function (estimated Glomerular Filtration Rate (eGFR) > 60 ml/min), resulted in a significant reduction in LVMi, albuminuria, and TKV increase, compared to standard blood pressure control (target 120–130/70–80 mmHg) (Chapman et al., 2010; Schrier et al., 2014). Similar conclusions were also reached in the HALT-B study that enrolled patients with more advanced kidney failure (eGFR 25–60 ml/min) (Torres et al., 2014). No significant differences were observed when comparing the efficacy of angiotensin converting enzyme inhibitor (ACE-i) monotherapy with a scale-up combination therapy with ACE-i and angiotensin II receptor blockers (ARBs), suggesting that benefits were related to achievement of a lower target blood pressure, rather than using a higher dose of blood pressure medications.

In 1993, Zeier et al. published one of the first pediatric studies on this subject, including 24 young ADPKD patients, 12 children (age 5.7–13.3 years) and 12 adolescents and adults (age 15.2–24.9 years), and 24 controls matched for age, sex and body surface area. All patients had normal kidney function. The authors studied left ventricular mass (LVM) by real-time directed M-mode echocardiography and observed a significantly higher LVMi across all ages without overt LVH. Blood pressure, measured by ABPM, was normal in children but was significantly higher in young adults, compared to controls. Left ventricular systolic function was normal in the two groups (Zeier et al., 1993).

In 1995, Ivy compared 83 ADPKD children with 66 unaffected siblings from 66 ADPKD families. A significantly higher prevalence of mitral valve prolapse (MPV) was found in affected patients compared to unaffected controls (12% vs. 3%). Patients with MPV were older and had more severe renal disease (>10 cysts). In addition, a significantly higher prevalence of hypertension was observed in affected children. In respect to LVMi, the authors observed a non-statistically significant trend towards higher values in ADPKD children (*p* = 0.07). When the LVMi was compared with systolic and diastolic blood pressure values in affected and unaffected children, a significant positive relationship between systolic blood pressure and LVMi (R = 0.43, *p* < 0.0001) was observed in ADPKD children. Hypertensive patients had significantly higher LVMi compared to normotensive patients. The latter group has LVMi values similar to normotensive controls (Ivy et al., 1995).

In 2008, Cadnapaphornchai assessed the impact of blood pressure in the clinical outcomes of 85 ADPKD children and young adults. Patients were divided into three groups according to their blood pressure status: hypertension (HBP, systolic and diastolic pressure higher than 95th percentile), borderline hypertension (BBP; 75–95th percentile), normotensive patents (NBP; less than 75th centile). The authors compared LVMi, TKV, microalbuminuria, and kidney function in these three groups. No significant differences were observed in kidney function and microalbuminuria. Compared to patients with normal blood pressure, both hypertensive and borderline hypertensive patients had higher LVMi. Of note, overt LVH was not present in any subgroup, but a statistically significant increase in LVMi was detected in patients with borderline pressure with still normal TKV (Cadnapaphornchai et al., 2008).

Data from the same cohort were subsequently published after 5 years of follow-up, during which patients received treatment with ACE-i. The RAAS blockade appeared to stabilize LVMi and prevent deterioration of kidney function in normotensive or borderline hypertensive ADPKD children, but not in patients that were hypertensive. The authors hypothesized that once hypertension was established, treatment with ACE-i alone may no longer be sufficient to prevent cardiovascular disease (Cadnapaphornchai et al., 2009).

Seeman et al. also observed higher average LVMi in children with ADPKD, compared to healthy controls [mean 30.4 ± 6.6 g/m2.7 vs. 26.2 ± 6.2 g/m2.7, *p* = 0.01], but no overt LVH (Seeman et al., 2021).

The above-cited studies have only analyzed the left ventricular geometry. Yet, LVM *per se* is not a measure of heart function and increased ventricular mass is not always associated with heart failure, although it is a contributing factor (Fick et al., 1995; Chapman et al., 1997; Ecder, 2013; Chebib et al., 2017; Chen et al., 2019; Kuo and Chapman, 2020). Very few data on cardiac function are available in ADPKD children.

In 1998, Bardaji et al. studied the ventricular function by trans-mitral pulsed Doppler flow and observed in addition to higher LVMi, evidence of early diastolic dysfunction (Bardaji et al., 1998).

**FIGURE 2:**
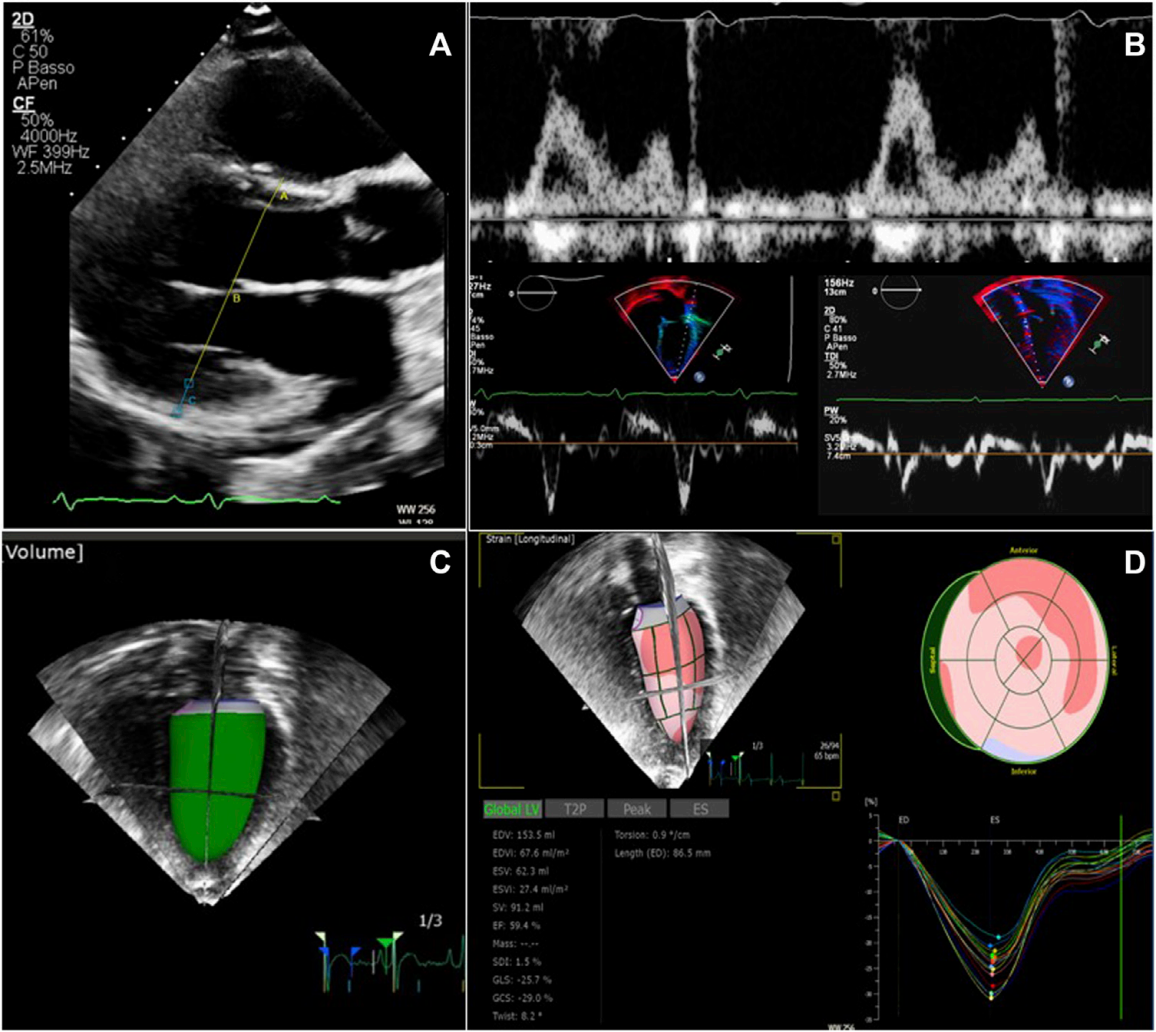
Echocardiographic examination in patients with ADPKD and ARPKD. **(A)** Left ventricular mass calculation from two-dimensional parasternal long axis view in end-diastole. **(B)** Diastolic function evaluation merging data from transmitral inflow velocities (upper image) and tissue Doppler velocities (lower images) from both the lateral and the septal left ventricular wall. **(C)** Real time three-dimensional left ventricular volume estimate **(D)** Global longitudinal strain analysis from speckle tracking imaging, using real time three dimensional acquisition.

Some illustrative images of advanced echocardiography are shown in Figure 2.

Another aspect of cardiovascular disease in ADPKD patients concerns arterial dysfunction. Endothelium dependent dilation (EDD), stiffening of the large elastic arteries, such as the aorta and carotid arteries, and increased carotid intima-media thickness (cIMT) are important independent predictors of cardiovascular events. In adult subjects with ADPKD, these parameters are abnormal, independently from the progression of kidney disease and before the onset of hypertension (Borresen et al., 2007).

Some studies have demonstrated that vascular dysfunction begins very early in the course of the disease, including during childhood. In 2017, Nowak et al., studied vascular dysfunction in 15 ADPKD children and young adults (age range 6–22 years) with normal kidney function, and in 15 controls. All subjects had normal blood pressure (<140/90 mmHg if adults or <95th percentile), but 8/15 (46%) patients with ADPKD were treated with ACE-is. The authors observed a significant decrease in the EDD in ADPKD subjects, as measured by brachial artery flow mediated dilation. The carotid-femoral pulse wave velocity (PWVcf), a measurement of arterial stiffness, was on average 14% higher in ADPKD children and young adults. The carotid augmentation index (cAIx) and carotid systolic blood pressure were also higher compared to the control group, while cIMT was similar (Nowak et al., 2017). In 2018, Karava et al. analyzed 21 ADPKD subjects aged 6–19 years and observed increased LVMi in 2 patients (9.5%), PWVcf in 4 patients (19%), and increased cIMT in 8 patients (38.1%). Anti-hypertensive therapy was not associated with higher PWVcf or cIMT (Karava et al., 2018).

Studies in young adults with ADPKD have also shown early vascular dysfunction independently from blood pressure values. Borresen et al. reported in 2007 that the reflection of the pulse wave was amplified in young normotensive ADPKD patients, demonstrating early arterial damage (Borresen et al., 2007). In 2009, Azurmendi et al. reported increased cIMT in patients with albumin/creatinine ratio >6.8 mg/g, regardless of the hypertension status. A linear correlation was observed between blood pressure levels and LVMi, but not with cIMT or PWVcf values (Azurmendi et al., 2009).

Marlais et al. compared in 2019 a large population of ADPKD children under the age of 18 with age-matched healthy controls. No significant differences in PWVcf were observed. This data differs from previous reports and differences are probably related to the younger age of patients in their cohort, suggesting that vascular changes develop only during late childhood and early adulthood. On average, children with ADPKD had higher blood pressure and LVMi, although overt LVH was not observed; 35% of patients lack nocturnal dipping (Marlais et al., 2019).

More recently, Seeman et al. studied the vascular function in ADPKD children and observed higher pulse pressure amplification in ADPKD subjects compared to controls, but no significant differences in the PWVcf (Seeman et al., 2021).

### 2.4 Final remarks

Taken together, current data indicate that the prevalence of hypertension in ADPKD children ranges 6%–47%. When considering only studies that have used ABPM to assess blood pressure, hypertension was observed in approximately one-third of patients. The main mechanism driving hypertension is the activation of the RAAS system, but other factors contribute to the elevation of blood pressure, including over-expression of endothelin receptors, increased synthesis of endothelin 1, sodium retention, and reduced synthesis of nitric oxide in the vascular endothelium. Most hypertensive children with ADPKD do not develop overt LVH, although their LVMi is on average higher compared to normotensive patients and to control subjects. Data on vascular dysfunction are scanty and findings are not uniform. They raise however, intriguing hypotheses on the pathogenesis of vascular damage in ADPKD that deserve further studies. Of note, children may be the ideal subjects to perform these studies because they may show early signs of vascular dysfunction independently from the development of hypertension.

Several limitations apply when analyzing the literature on ADPKD in children in respect to the prevalence of arterial hypertension. The majority of studies are retrospective. The size and age of cohorts are often very different. Most studies were carried out in third-level centers that tend to recruit more severe patients and more motivated families. This may cause overestimating the prevalence of hypertension and cardiovascular consequences. In addition, the methodologies used to assess blood pressure and cardiovascular damage vary in different studies.

Nevertheless, the available data support regular measurement of blood pressure once a year in children with ADPKD or at risk of ADPKD, as suggested by expert opinion publications (Gimpel et al., 2019). Measuring blood pressure is minimally invasive and should be part of the routine pediatric check-up. These measurements can be willingly accepted by children of affected patients that have not yet undergone diagnostic procedures to rule out ADPKD. Although the vast majority of ADPKD children do not have LVH, one can hypothesize that early initiation with anti-hypertensive therapies may limits future development of cardiovascular organ damage. It is unclear if this will also modify the evolution of cystic disease. The European Pediatric Registry on Polycystic Dominant Kidney (ADPedKD Registry) may in the future answer this question. In other chronic kidney diseases, pediatric nephrologists have learned that early treatment of hypertension may slow down the progression of CKD and reduce cardiovascular complications. In this respect, pediatricians may be in a privileged position to impact significantly on the outcome of ADPKD patients by acting very early during the course of the disease.

## 3 Autosomal recessive polycystic kidney disease (ARPKD)

### 3.1 Introduction

Although ARPKD is a rare disease, it is the foremost cystic disease in early childhood. This ciliopathy is caused by biallelic variants of the *PKHD1* gene that encodes for the nephrocystin protein. In the kidneys, nephrocystin localizes to the sensory *cilium* in the cortical and medullary collecting ducts and thick ascending limbs of the loop of Henle. In addition, nephrocystin is expressed in the biliary and pancreatic tracts. Compared to ADPKD, recessive inheritance causes early onset and more severe symptoms, although significant phenotypic variability is observed. Severe perinatal disease has been associated with biallelic null variants, but biallelic missense variants do not exclude very early onset of a severe disease. A recent genotype-phenotype correlation study including 304 children with ARPKD has shown that the location of variants in different domains of the *PKHD1* gene plays an important role in determining the phenotype (Burgmaier et al., 2021).

The two organs involved in ARPKD are the kidney and the liver. The latter usually present with congenital hepatic fibrosis, which is sometimes associated with Caroli disease. Enlarged kidneys with non-obstructive fusiform dilations of collecting ducts are the main characteristics of renal involvement. Since the expression of the disease begins very early, most cases are identified *in utero* or immediately after birth. Kidney failure may develop prenatally causing oligohydramnios, which in turn impairs normal lung development. Pulmonary hypoplasia in newborns with severe antenatal ARPKD is the main cause of death in the first year of life (Roy et al., 1997; Guay-Woodford, 2014; Guay-Woodford et al., 2014). However, clinical expression is extremely variable in terms of severity and age of onset of symptoms, for both renal and hepatic phenotype.

Early diagnosis and improvements in neonatal intensive care have significantly increased the survival rate of patients with early clinical expression. Non-etheless, 30%–40% of patients still die from pulmonary hypoplasia and respiratory failure (Kaplan et al., 1989; Deget et al., 1995; Roy et al., 1997; Rhona Capisonda et al., 2003; Guay-Woodford et al., 2014). Perinatal survival rates are also be influenced by medical and/or parental decisions to withhold treatment in oliguric infants with severe pulmonary distress. Patient survival increases markedly after the first month of life (Bergmann et al., 2005; Dell, 2011), in particular regarding patients who survived to 1 year of age, 82% of them were alive at 10 years (Kaplan et al., 1989; Bergmann et al., 2005). Historical data reported conflicting results on the correlation between kidney size and kidney function. In 2021, data from the European Recessive Polycystic Kidney Registry (ARegPKD) demonstrated an inverse correlation between TKV adjusted for length in the first 18 months of life and kidney survival from prenatal life to adolescence (Burgmaier et al., 2021b).

### 3.2 Pathogenesis and prevalence of hypertension

As for ADPKD, hypertension is a main symptom of ARPKD that on average develops much earlier. The estimated prevalence of hypertension in published studies ranges from 33% to 75% (Deget et al., 1995; Zerres et al., 1996; Guay-Woodford and Desmond, 2003; Bergmann et al., 2005; Dell, 2011; Guay-Woodford et al., 2014). Hypertension develops even in the first weeks of life and is often difficult to control, requiring early combined therapy with different antihypertensive drugs (Deget et al., 1995; Zerres et al., 1996; Guay-Woodford et al., 2014; Chinali et al., 2019; Seeman et al., 2022). Hypertension is not always associated with kidney failure. In patients with preserved kidney function, it usually precedes the development of CKD (Zerres et al., 1996; Guay-Woodford and Desmond, 2003).

In 1987, Cole et al. were among the first investigators to describe high prevalence of severe early-onset hypertension in patients with ARPKD (Cole et al., 1987). At that time, the disease was not very well studied. In the following years, several publications have studied the outcome of children with ARPKD and have reported high prevalence of arterial hypertension, although blood pressure was not a primary outcome measurement of these investigations.

In 1989, Kaplan et al. observed a 65% prevalence of hypertension in 55 patients with ARPKD. In the same study, they monitored plasma renin and serum sodium levels, and concluded that in ARPKD, arterial hypertension is probably not driven by renin but by an increase in intravascular volume, as suggested by hyponatremia, which they observed frequently, especially in younger children (Kaplan et al., 1989). A few years later, Deget et al. reported a similar prevalence of hypertension, but observed that the age of onset was extremely variable, ranging from the first months of life to adolescence (Deget et al., 1995).

Soon after, Zerres et al. published a multicenter study in which 115 patients were recruited in 34 pediatric nephrology centers. More than half of the cohort (i.e., 77/115 children) needed antihypertensive treatment during the observation period, and 50% were hypertensive at diagnosis. Overall, that study showed a very high prevalence of early-onset hypertension; 64% of patients had started anti-hypertensive therapy within the first year of life. At the last observation, one-third of patients had not achieved satisfactory blood pressure control (Zerres et al., 1996).

In 1997, Roy et al. reported somewhat lower rates of early-onset hypertension in another cohort followed from the first month of life. In their cohort, the percentage of patients requiring antihypertensive therapy increased from 39% at 1 year of age to 60% at the age of 15 years (Roy et al., 1997). A higher prevalence was observed by Rhona Capisonda et al., who studied 31 ARPKD patients aged 0–14 years at diagnosis. When first evaluated, 55% of patients were hypertensive. The prevalence increased to 85% in patients who had survived the neonatal period, and approximately half of patients needed more than one medication to achieve adequate blood pressure control. The age of onset of hypertension ranged 4 days to 3 years. Interestingly, the pharmacological needs to achieve blood pressure control seemed to decrease over time, even in patients with very early-onset hypertension, a finding that indirectly emerges also from the analysis of other studies. Similarly to the data reported by Kaplan et al., the authors described in 3 infants a positive association between hyponatremia and hypertension (Rhona Capisonda et al., 2003). Hyponatremia is a frequent finding in ARPKD children. It is not associated with increased urinary sodium losses and develops before progression to CKD, probably as a consequence of altered free-water clearance and possibly increased sodium reabsorption, resulting in hypervolemia, especially in infants who are exposed to a lower osmotic load (Kaplan et al., 1989; Zerres et al., 1996; Guay-Woodford and Desmond, 2003; Guay-Woodford et al., 2014; Wicher et al., 2021).

In 2003, Guay-Woodford et al. published data from the North American ARPKD Clinical Database. The study included 209 patients stratified by year of birth; 21% and 79% of patients were born before and after 1990, respectively. The prevalence of hypertension and the risk of developing hypertension were higher in patients born before 1990 (80% vs. 65%). The interpretation of this finding is unclear and may be altered by biases. The median age at diagnosis was significantly lower in more recent patients, most likely reflecting improvements in the diagnosis and care of sick newborns and infants over the years. The age of onset of hypertension and of CKD was significantly lower in infants requiring mechanical ventilation, reflecting more severe disease. In patients born after 1990 that survived the first month of life, the age of onset of hypertension and of CKD were positively correlated, although this finding does not necessarily imply a direct cause-and-effect relationship between these two variables. In addition, 96% of patients with early-onset hypertension had hyponatremia, supporting the hypothesis that hypertension in ARPKD may be the result of a dysregulation in the sodium reabsorption mechanism in the ectatic collecting ducts, but they also recognize that this mechanism has not been demonstrated by other studies (Guay-Woodford and Desmond, 2003).

In a 6-year follow-up study published by Bergmann et al., in 2015 to assess genotype-phenotype correlations, the prevalence of hypertension was 43% at the beginning of observation period and 73% at the end of follow-up. Anti-hypertensive treatment was started at an average age of 3 years; half of patients were receiving treatment at the age of 6 months (Table 2) (Bergmann et al., 2005).

As for ADPKD, most studies in ARPKD children have assessed hypertension with office blood pressure measurements. More recently, ABPM has also been used in children with ARPKD. Seeman et al. have published a retrospective study on 36 children with ARPKD, of whom 29 had performed at least two ABPM recordings. The median age at the first ABPM was 4.4 years and the average interval between the first and the last recording was 5 years. Confirming previous reports (Dell et al., 2016), the initial evaluation showed hypertension in 94% of patients, of which only one-third were well controlled with antihypertensive therapy (Seeman et al., 2022). At the last evaluation, the prevalence of hypertension remained stable (86%), but two-third of patients were now adequately treated. Blood pressure did not correlate with kidney length nor with glomerular function. The prevalence of hypertension was similar when using ABPM or OBPM (94% and 86%), probably because values were markedly elevated with both methods. The improvement of blood pressure control overtime may be related to physician tendency to use more aggressive blood pressure therapy in older children, but may also reflect a natural tendency of blood pressure to improve spontaneously in older children with ARPKD (Rhona Capisonda et al., 2003).

The pathophysiology of hypertension in ARPKD is still incompletely understood. Volume overload secondary to CKD may represent the main factor driving blood pressure increase (Kaplan et al., 1989; Rhona Capisonda et al., 2003). In 2005, Rohatgi et al. have analyzed the fluid composition of renal cysts obtained from a murine model of ARPKD and from nephrectomized kidneys of patients with ARPKD. They observed increased reabsorption of sodium in murine epithelial cells, but not in human cells (Rohatgi et al., 2005). Some years after, data from the ARPKD mouse model suggest a local paracrine activation of the intra-renal RAAS mediated by up-regulation of RAAS genes in immature tubular structures, without significant systemic activation (Goto et al., 2010). Additional hypotheses include up-regulation of the epidermal growth factor/epidermal growth factor receptor axis, activation of non-classic components of the RAAS (angiotensin 1-7, ACE2 and Angiotensin II type 2 receptor) and increased cyclic adenosine monophosphate (cAMP) activity, which, in addition to its role in cystogenesis, can stimulate overexpression of RAAS genes (Goto et al., 2010).

Despite several studies have reported low circulating renin levels in children with ARPKD, RAAS inhibitors are the most frequently medications used to treat hypertension and are efficient in most patients.

### 3.3 Cardiovascular consequences of hypertension

Studies on cardiac involvement in ARPKD children are very limited and data are not homogeneous due to differences in the techniques that have been used to assess cardiac geometry and function.

In 2007 Phillips et al. have studied cardiac geometry in an ARPKD mouse model (Lewis polycystic kidney) in which kidney cysts develop from the age of 3 weeks, hypertension at 6 weeks of age, and LVH at 24 weeks of age, indicating a clear temporal sequence between progression of kidney disease, hypertension, and cardiac damage (Phillips et al., 2007).

Dell et al. have studied the cardiac geometry as a secondary outcome measure in pediatric patients with ARPKD and children with mild-to-moderate CKD secondary to renal dysplasia and urinary tract obstruction. No significant differences in the prevalence of LVH and hypertension (defined with ABPM) were observed. However, a significantly greater percentage of ARPKD children received blood pressure medications compared to children with other renal diseases (Dell et al., 2016).

Seeman et al. have also evaluated cardiac geometry in ARPKD children using two-dimensional echocardiography. They observed LVH in 30% of patients, including some patients that had good blood pressure control (Seeman et al., 2022).

We have studied 27 ARPKD children at an initial age of 3.8 years. Of these, 92% were treated with antihypertensive drugs, which had been started before 6 months of life in 48% of patients. Compared to age- and sex-matched healthy children, patients with ARPKD had on average significantly higher LVMi and showed a significantly higher prevalence of LVH, mainly of the concentric type (Chinali et al., 2019). Multivariate analysis showed a significant association between LVMi and blood pressure, confirming the predominant role of arterial hypertension in inducing LVH (Mitsnefes et al., 2016). We also analyzed the ejection fraction (EF), the longitudinal strain, the circumferential strain, and the midwall fractional shortening (FSmw). These latter parameters allow identifying geometry independent subclinical systolic dysfunction (Narayanan et al., 2009). The EF was normal in children with ARPKD, but 22% had abnormal FSmw and/or circumferential strain values, indicating subclinical systolic dysfunction. Of note, FSmw did not correlate with blood pressure, with the EF and with kidney function. ACE-i/ARBs were weakly associated with improvements in FSmw over time, supporting the use of these drugs to treat myocardial dysfunction and suggesting a non-hemodynamic effects of the RAAS system on cardiac remodeling, as already proposed by others (Schlaich and Schmieder, 1998).

Some illustrative images of advanced echocardiography are shown in Figure 2.

Altogether and despite the small number of patients included in the few available studies, current data indicate that children with ARPKD have significantly higher LVMi and develop concentric LVH in a significant proportion of cases. In addition, many have evidence of subclinical cardiac dysfunction, similarly to children with CKD secondary to other causes (Chinali et al., 2015), but differently form adult hypertensive subjects that for the most part develop selective alteration of the longitudinal strain (Narayanan et al., 2009; Sengupta et al., 2013). Incomplete correlation between cardiac abnormalities and blood pressure values suggest that LVH in ARPKD may not be driven solely by arterial hypertension.

### 3.4 Final remarks

Compared to ADPKD, ARPKD is a much rarer disease. Therefore, fewer data are available on cardiovascular consequences. Furthermore, younger age and more severe disease of patients at diagnosis renders more difficult assessing blood pressure using ABPM and performing sophisticated cardiac investigations. Overall, severe, early-onset hypertension is more frequent in ARPKD children. The prevalence of hypertension ranges 55%–100% in different studies. The pathogenesis of hypertension is not fully elucidated and may not be entirely due to the activation of the RAAS. However, ACE-i and ARBs remain the mainstay of treatment, together with calcium channel blockers. Frequently, more than one drug is needed to treat hypertension, although blood pressure control tends to improve spontaneously over time. Approximately one-third of patients surviving the neonatal period have abnormal cardiac geometry. More studies are needed to establish the role of genotype in the severity of cardiovascular changes.

## 4 Conclusion

ARPKD and ADPKD are severe ciliopathies that are characterized by marked cardiovascular involvement. In both diseases, early detection of hypertension and of cardiac anomalies allows limiting cardiovascular damage and long-term complications. To which extent very early treatment of cardiovascular symptoms impact on the evolution of kidney diseases remains uncertain but deserves more attention in the future. In the meantime, pediatricians can play a crucial role in balancing prevention and excessive medicalization, in particular in children with ADPKD.
